# Enterococcal colonization of the gastro-intestinal tract: role of biofilm and environmental oligosaccharides

**DOI:** 10.1186/1471-2180-6-60

**Published:** 2006-07-11

**Authors:** Roberta Creti, Stefanie Koch, Francesca Fabretti, Lucilla Baldassarri, Johannes Huebner

**Affiliations:** 1 Channing Laboratory, Brigham and Women's Hospital, Harvard Medical School, Boston, MA, USA; 2 Dipartimento di Malattie Infettive, Parassitarie ed Immunomediate – Istituto Superiore di Sanità, Roma, Italy; 3 Division of Infectious Diseases, Department of Medicine, University Hospital Freiburg, Germany

## Abstract

**Background:**

Biofilm formation in *E. faecalis *is presumed to play an important role in a number of enterococcal infections. We have previously identified a genetic locus provisionally named *bop *that is involved in maltose metabolism and biofilm formation. A transposon insertion into the second gene of the locus (*bopB*) resulted in loss of biofilm formation, while the non-polar deletion of this gene, together with parts of the flanking genes (*bopA *and *bopC*) resulted in increased biofilm formation. A polar effect of the transposon insertion on a transcriptional regulator (*bopD*) was responsible for the reduced biofilm formation of the transposon mutant.

**Results:**

The amount of biofilm formed is related to the presence of maltose or glucose in the growth medium. While the wild-type strain was able to produce biofilm in medium containing either glucose or maltose, two mutants of this locus showed opposite effects. When grown in medium containing 1% glucose, the transposon mutant showed reduced biofilm formation (9%), while the deletion mutant produced more biofilm (110%) than the wild-type. When grown in medium containing 1% maltose, the transposon mutant was able to produce more biofilm than the wild-type strain (111%), while the deletion mutant did not produce biofilm (4%). Biofilm formation was not affected by the presence of several other sugar sources. In a gastrointestinal colonization model, the biofilm-negative mutant was delayed in colonization of the mouse intestinal tract.

**Conclusion:**

The biofilm-positive phenotype of the wild-type strain seems to be associated with colonization of enterococci in the gut and the presence of oligosaccharides in food may influence biofilm formation and therefore colonization of enterococci in the gastrointestinal system.

## Background

**Figure 1:**
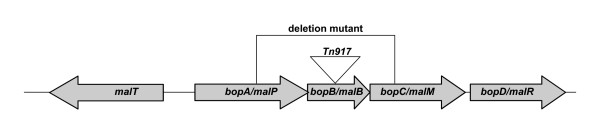
Schematic representation of the Bop locus (modified from 8 and 10).

Enterococci are important causes of hospital-acquired infections, and treatment of infections due to these opportunistic pathogens is becoming increasingly difficult because of their resistance to multiple antibiotics [1]. The clinical importance of biofilm formation has been proposed for several enterococcal infections, but the information regarding the basic molecular mechanisms is fragmentary [2-6]. We have recently identified a four-gene locus involved in biofilm formation in *Enterococcus faecalis*, provisionally named *bop *(biofilm on plastic surfaces, see Figure 1). Recently, Bourgogne and coworkers confirmed that genes of this locus are regulated by the Fsr system, a quorum-sensing mechanism involved in the expression of virulence genes in *E. faecalis *[7]. A putative sugar-binding transcriptional regulator, *bopD*, was found to be necessary for biofilm formation. The transposon insertion occurred into the second gene of the operon, but the reduction of biofilm formation is related to a polar effect on the 4. gene (i.e. the sugar-sensing transcriptional regulator *bopD*). Complementation of the transposon mutant was done using all the separate genes (i.e., *bopA*, *bopB*, *bopC*, and *bopD*) with only the *bopD *gene being able to partially restore biofilm formation [8]. We thus concluded that this gene may be important to integrate nutritional cues, such as the availability of certain carbohydrates, into the signal transduction pathway regulating biofilm expression and probably other virulence mechanisms [8,9]. Since *bopD *exhibits significant sequence homology with a number of bacterial proteins involved in the regulation of maltose metabolism [10], we evaluated the biofilm formation in medium containing glucose or maltose of the wild-type (*E. faecalis *T9) and two mutants, a biofilm-negative transposon mutant (*E. faecalis *10D5 with insertion of the transposon into the *bopB *gene) and a biofilm-enhanced deletion mutant (*E. faecalis *TDM with deletions of parts of *bopA *and *bopC *and all of *bopB*). The possibile effect of oligosaccharide in the diet on the colonization and biofilm production of resident enterococci in mice was also evaluated.

## Results

### Biofilm formation of the *E. faecalis *strains grown in glucose or maltose medium

**Figure 2:**
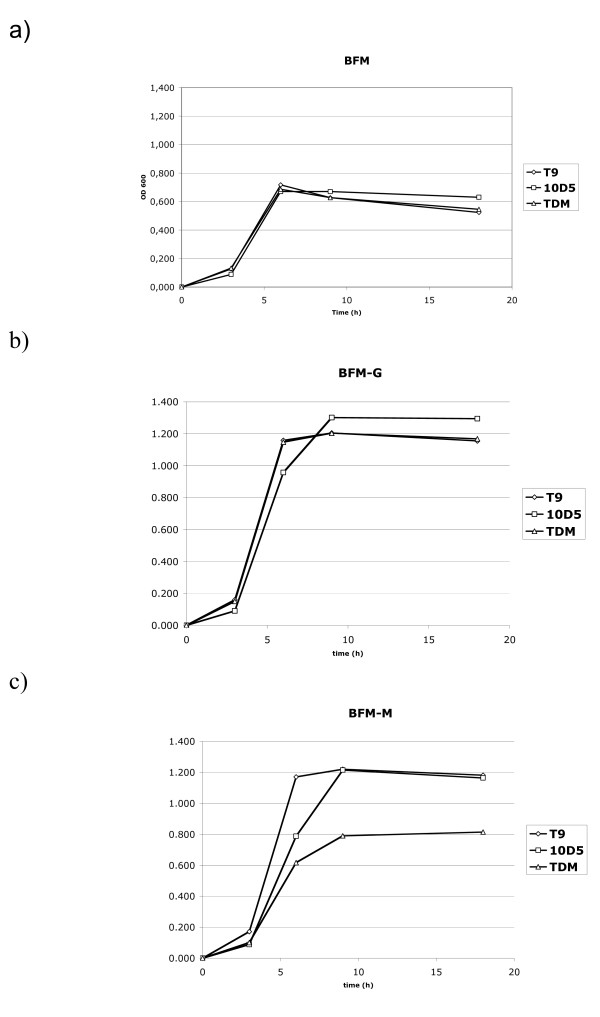
Growth curves of *E. faecalis *T9 (diamonds), 10D5 (squares), and TDM (triangles) in BFM (a), BFM-G (b), and BFM-M (c). The OD_595 _after 18 h of incubation was comparable for the different strains in medium without additional sugars and in medium supplemented with 1% glucose. The wild-type strain and the transposon mutant grew equally well in medium containing 1% maltose, while the triple deletion mutant grew significantly less.

The optical density measured in the microtiter plates after 18 h of incubation was not statistically different for the various strains (*E. faecalis *T9, *E. faecalis *10D5, and *E. faecalis *TDM) grown in medium without additional sugars and in medium supplemented with 1% glucose (BFM-G) (Figure 2A and 2B). In biofilm medium supplemented with 1% maltose (BFM-M), the wild-type strain (*E. faecalis *T9) and the transposon mutant (*E. faecalis *10D5) grew equally well, while the triple deletion mutant (*E. faecalis *TDM) grew significantly less than the other two strains (Figure 2C). None of the strains produced biofilm in medium without sugar or in medium that contained 0.25% glucose or 0.25% maltose (data not shown).

**Figure 3:**
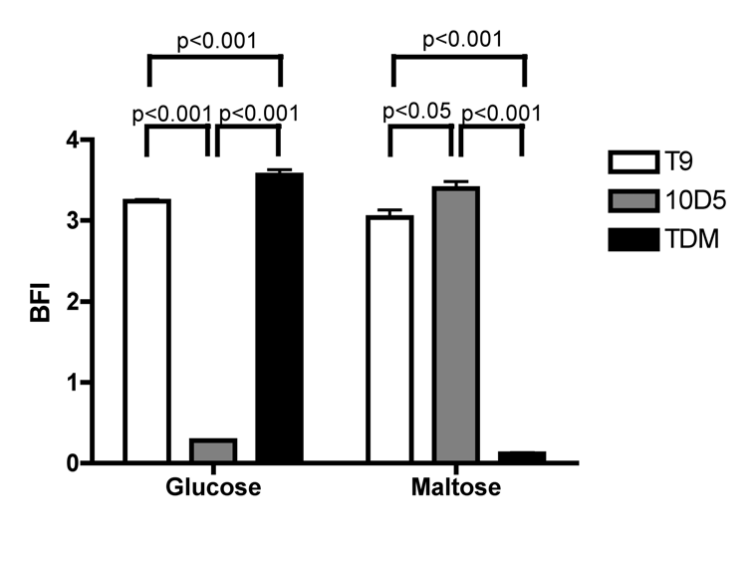
Biofilm formation of the wild-type and the two mutants in glucose (a) or maltose-containing medium (b). Biofilm formation by the wild-type was similar in medium containing glucose or maltose. The biofilm-negative transposon mutant E. faecalis produced significantly less biofilm in medium containing 1% glucose but equal amounts to the wild-type when grown in 1% maltose. The deletion mutant produced significantly more biofilm than the wild-type strain in medium containing glucose but significantly less biofilm than the wild-type or the transposon mutant E. faecalis when grown in medium containing 1% maltose.

**Figure 4:**
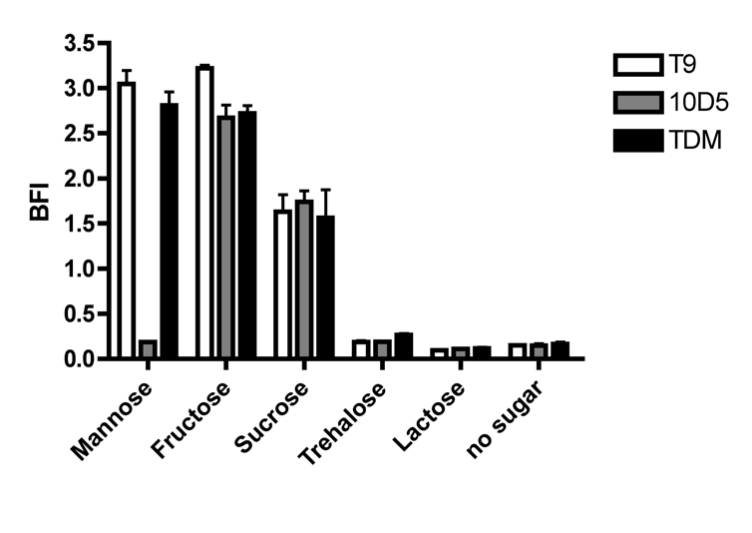
Biofilm formation of the wild-type and the two mutant strains in medium containing various sugars. Addition of mannose and resulted in no biofilm production by the transposon mutant and strong biofilm production by the wild-type and the deletion mutant TDM. All strains produced strong biofilm when 1% fructose was added to the medium. Biofilm formation was reduced in 1% sucrose fro all strains and no biofilm was seen in medium containing trehalose or lactose.

Biofilm formation by the wild-type *E. faecalis *T9 did not differ in glucose- and maltose-containing medium (Figure 3). As shown before [8], the biofilm-negative transposon mutant *E. faecalis *10D5, when grown in BFM-G, produced significantly less biofilm than the wild-type (Figure 3a). However, when the bacteria were grown in BFM-M (Figure 3b), the wild-type *E. faecalis *T9 and the transposon mutant *E. faecalis *10D5 produced equal amounts of biofilm. When grown in BFM-G, the deletion mutant *E. faecalis *TDM produced significantly more biofilm (127%) than did the wild-type strain *E. faecalis *T9 (Fig. 3a). However, when this mutant was grown in medium containing maltose as the single carbon source, it produced significantly less biofilm (24%) than did the wild-type *E. faecalis *T9 or the transposon mutant *E. faecalis *10D5. As expected, all strains produced biofilm in medium containing both 1% glucose and 1% maltose (data not shown). We also tested biofilm formation of the wild-type strain and the two mutant strains in medium containing various sugars (Figure 4) and observed that mannose and glucose produced similar activity (i.e., no biofilm production by the transposon mutant 10D5 and strong biofilm production by the wild-type and the deletion mutant TDM). All strains were able to form strong biofilm when fructose was added to the medium, while biofilm formation was reduced in 1% sucrose (see Fig. 4). No biofilm was observed for any strains in cultures containing trehalose or lactose.

### Scanning electron microscopy

To obtain a comparable view of the biofilm composition, micrographs were taken from randomly chosen fields at the same magnification for each sample.

**Figure 5:**
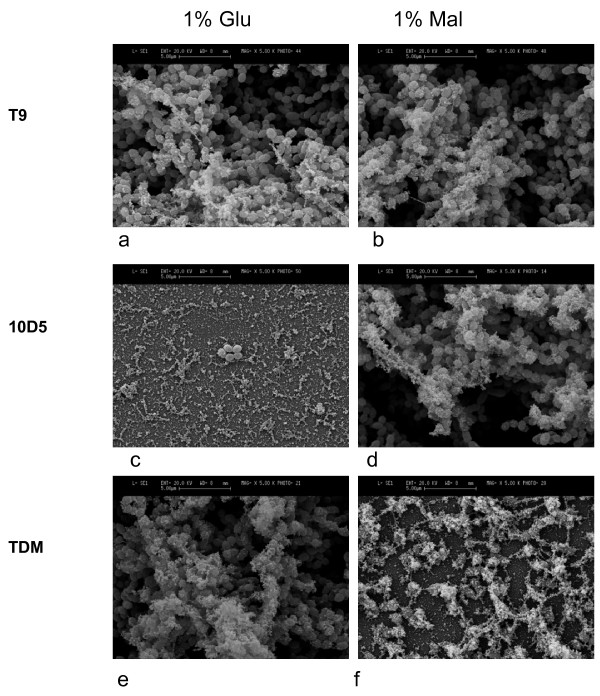
Scanning electron micrographs of the wild-type and the two mutants grown in 1% maltose or 1% glucose. The wild-type formed a well-organized, multilayered biofilm in all growth conditions. The transposon mutant formed biofilm only in maltose while the triple deletion mutant exhibited a well-formed biofilm in glucose. Maltose induced the formation of amorphous material covering the cells. Biofilm in glucose and maltose was different: wild-type bacteria grown in glucose showed small aggregates on the cell surface and these aggregates became larger and less regular when bacteria were grown in maltose. These larger aggregates where also present on the surface of the transposon mutant when grown in maltose and on the deletion mutant grown in glucose.

T9 appeared to form a well-organized, multilayered biofilm in all growth conditions (Fig. 5a and 5b), while the transposon mutant 10D5 formed biofilm only when maltose was present (Fig. 5c and 5d). The triple deletion mutant exhibited a well-formed biofilm in glucose (Fig. 5e), while maltose appeared to induce the formation of large aggregates of amorphous material apparently covering the cells (Fig 5f); in this case, however, no multilayered accumulation of cells was observed.

Biofilm formed in glucose and maltose appears to differ slightly: T9 cells grown in glucose presented small globular aggregates on the cell surface (Fig. 5a) that became larger and less regular when bacteria were grown in maltose (Fig. 5b). Larger aggregates where also visible on the surface of 10D5 grown in maltose (Fig. 5d) and on the deletion mutant grown in glucose (Fig 5e).

Extracellular material visible on scanning electron micrographs represents polysaccharides partially collapsed due to dehydration caused by processing for SEM. However, we always examined samples processed in a single run, to avoid biased observation caused by artifacts; the differential "collapse" of the extracellular material when bacteria were grown in glucose or maltose, suggests that the material does in fact differ.

### Mouse colonization model

**Figure 6:**
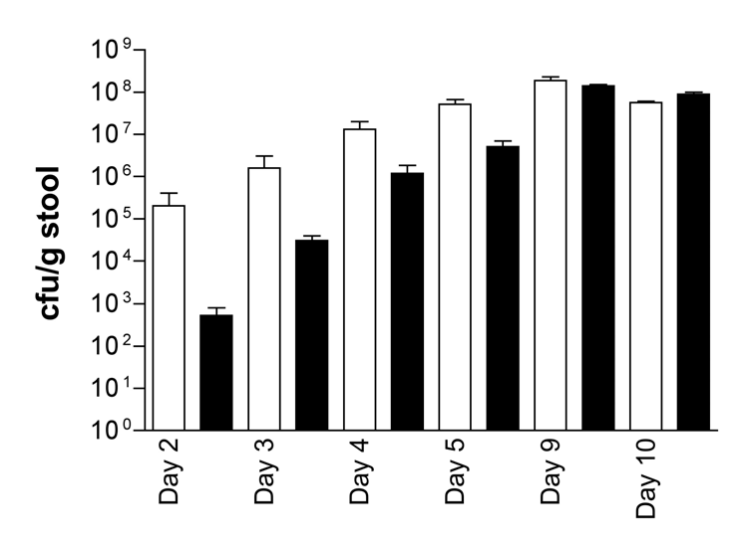
Gastrointestinal colonization of mice with the wild-type (white bars) or the biofilm-negative transposon mutant 10D5 (black bars). Colonization of mice with the transposon mutant was delayed with about 5–10 times reduced colony counts during the first 5 days of the experiment. After that the colony counts were comparable to the wild-type strain.

Mice were treated with oral antibiotics to eliminate their physiological gastrointestinal flora. After bacterial challenge with different strains in the drinking water the animals showed increasing gastrointestinal colonization over a period of 9 days and stable colony counts in the stool thereafter (data not shown). The transposon mutant resulted in a delayed colonization with about 5–10 times reduced colony counts during the first 5 days of the experiment. After that the colony counts were comparable to the wild-type strain (see Figure 6).

## Discussion

Enterococci are important inhabitants of the gastrointestinal tract of humans and many animals [11]. While a number of colonization and adhesion factors have been studied, the ability of enterococci to effectively colonize the gut is not well understood. Several studies have investigated biofilm formation of enterococci, which is thought to be a multifactorial event [6,8,12-16].

Le Breton and colleagues identified the locus described by us previously [8] as being responsible for the uptake and metabolism of maltose [10]. From these data and from our results it seems clear that *bop*D is likely to be a Maltose-sensitive negative regulatory protein that may repress both *bopABC*, and the divergently transcribed *malT *operon. Two insertional mutants were studied by Le Breton et al., one in the *malT *and one in *bopA *(named *malP *by Breton et al.). These results confirm the role of this locus in the utilization of maltose and corroborate our observations with regard to the growth curves in our mutants. *E. faecalis *TDM shows significantly decreased growth in medium containing maltose as the single sugar source compared with the other strains (T9 and 10D5). However, the ability to form biofilm seems to be independently reduced under these conditions because all the optical densities measured for the biofilm formation were normalized to take into account the different growth rates.

Trehalose and maltose are abundant disaccharides in nature and serve as important carbon and energy sources to lactic acid bacteria. Maltose is generated by enzyme-catalyzed hydrolysis of starch by amylases present in the gastrointestinal tract. In *L. lactis *the genes encoding maltose phosphorylase (MP), trehalose-6-phosphate phosphorylase (TrePP), and β-phosphoglucomutase are induced by maltose and trehalose, indicating that the trehalose and maltose catabolic pathways are closely connected [17,18]. However, when our wild-type and mutants were grown in medium containing 1% trehalose, the effect on growth was different with respect to growth in maltose and no biofilm was formed (see Figure 4).

It has been observed that, in the hyperthermophilic bacterium *T. maritima*, at high growth rates, maltose consumption increased significantly, although it appeared that carbon was used in the formation of extracellular polysaccharide (EPS) rather than accumulation of biomass [19]. The authors speculated that EPS formation could reflect the processing of excess carbon or, alternatively, could be coupled to a specific ecological strategy, such as biofilm formation.

It has been hypothesized that whenever bacteria are in non-optimal growth conditions (such as excess of carbon sources in the environment and/or altered sugar-metabolizing gene expression), the accumulation of reducing equivalents can be disposed of through the production of biofilm to transport these molecules out of the cell. In fact, it has been proposed that bacteria form exopolysaccharide matrices as a by-product to release reducing equivalents that could otherwise function as a bottleneck in the metabolism of an excess of the carbon source [19,20].

The observed effects of reduced biofilm formation in the transposon mutant grown in glucose could be attributable to the lower expression of the BopD protein and its subsequent minor efficiency in carbon catabolite regulation.

On the other hand, lower expression of *bopD *results in derepression of the *bopA *gene (a maltose phosphorylase), and to a minor extent also of the upstream sugar transport gene *malT*, as confirmed by real-time PCR (data not shown), which in turn may lead to a higher efficiency in transport and maltose utilization and enhanced biofilm formation ability when bacteria are grown in maltose.

In the deletion mutant TDM, the transcription of the *bopD *gene seems to be somewhat enhanced with respect to the wild-type. This probably results in increased repression of the transcription of *bopA *compared to the wild-type and the transposon mutant 10D5, as confirmed by real-time PCR (data not shown). However, *E. faecalis *TDM lacks functional BopA, BopB (a phosphoglucomutase), and BopC (an aldose-1-epimerase). Consequently, when this strain is grown in BFM-M, it may not be able to use maltose to produce the extracellular macromolecules necessary for biofilm formation, while its ability to metabolize glucose may not be affected.

Moreover, when *E. faecalis *TDM is grown in glucose, the overexpression of *bopD *could increase alternative pathways for glucose metabolism and in turn lead to enhanced biofilm formation.

In the gastrointestinal colonization model, we could demonstrate that the biofilm-negative mutant was delayed in colonizing the mouse intestinal tract, although the levels achieved after 9 days were eventually as high as for the wild-type strain. However, since the transposon mutation also leads to different expression of other genes putatively involved in biofilm formation [9], these experiments cannot completely rule out pleiotropic effects that may be responsible for the observed differences. Using stringent decolonizing methods in this experiment that provide a very artificial "mono-organism" colonization, interactions of these strains with other organisms cannot be ruled out.

A mechanism that integrates the availability of certain carbohydrates into the signal transduction pathway regulating biofilm expression could be important for the ability of enterococci to colonize the gastro-intestinal system of many animals and humans. Biofilm formation could help the bacteria adhere to the gut wall and may represent an advantage for certain strains. The formation of a biofilm seems to be related to a multicellular architecture and copious amounts of extracellular macromolecules, as shown in the scanning electron micrographs. This organization depends on the presence of specific genes and specific carbohydrate pathways as well as specific oligosaccharides in the environment.

## Conclusion

Although the functional role of the above-mentioned mechanism needs to be further elucidated, we speculate that the availability of starch and maltose in food and gastrointestinal contents may influence the expression of biofilm by enterococci and that this biofilm formation may enable these bacteria to colonize and persist in the gut.

## Methods

### Bacterial strains

The *E. faecalis *bacterial strains and mutants used in the present study are shown in Table 1. A rich medium that contained no additional carbohydrate source was used to study biofilm formation (BFM; 17 g of pancreatic digest of casein, 5 g of NaCl, 3 g of yeast extract, and 2.5 g of dipotassium phosphate per liter). Filter-sterilized oligosaccharides (all from Sigma Chemicals; glucose: BFM-G, maltose: BFM-M, as well as mannose, fructose, trehalose, lactose, and sucrose) were added to a final concentration of 1% (w/v), and bacteria were inoculated from overnight liquid cultures (primary culture).

### Biofilm assay

The primary culture was diluted 1:10 into polystyrene tissue culture-treated microtiter plates (Corning, Corning, NY) and grown at 37°C for 18 h. Growth was measured spectrophotometrically (OD_595 nm_); the plates were emptied, washed three times with PBS, and dried at 60°C for 1 h. The biofilm was subsequently stained for 2 min with Hucker's crystal violet [13]. The plates were washed thoroughly with tap water and dried, and the OD was measured with an ELISA reader at 595 nm. Biofilm formation was normalized to growth with the biofilm index (BFI), which was calculated as OD_biofilm _× (0.5/OD_growth_) [21]. Multigroup comparisons were made by ANOVA with Tukey's multiple comparison test using the Prism3 software package.

### Scanning electron microscopy

For biofilm formation, inocula were prepared in BFM-M and BFM-G, which were used to inoculate 24-well tissue culture plates (Costar, Corning Inc., Corning, NY) containing segments of polystyrene and BFM-M or BFM-G. Biofilm formed on polystyrene pieces was fixed according to Fassell et al. [22] for best preservation of polysaccharides. Briefly, samples were pre-fixed for 20' with Na-cacodylate-1% glutaraldehyde, supplemented with lysine 75 mM and ruthenium red 0.075% (w/v). Samples were then treated with Na-cacodylate-1% glutaraldehyde supplemented with ruthenium red 0.075% (w/v) for 1 hour at room temperature, and OsO4 1% for 1 hour. Dehydration with graded series of ethanol solutions was followed by critical point drying, gold sputtering, and observation with a Cambridge SE 360 scanning electron microscope.

### Mouse colonization with *E. faecalis *wild-type and mutant strains

Female BALB/c mice (Harlan-Sprague Dawley, Inc.) were kept in groups of 4 mice in cages with microisolator tops. The bedding (alpha chip, Northeastern products corporation, Warrensburg, NY), cages, and drinking bottles were autoclaved and changed every other day. Mice were fed irradiated mouse chow (PicoLab mouse diet 20, #5038, from PMI Nutrition International, Inc., Brentwood, MO). The drinking water, regular tap water, was autoclaved and supplemented with 1 g/l vancomycin hydrochloride (for intravenous use, Novaplus, Abbott Laboratories, North Chicago, IL), 100 mg/L metronidazole (Sigma), 1 g/L gentamicin (Sigma), and 1 g/L cefoxitin (Baxter Healthcare Corp., Syracuse NY). Mice were kept on this decolonizing antibiotic regimen for 10 days. Fecal pellets (1 per mouse) were collected every other day, weighed, homogenized in 750 μl Todd-Hewitt-Broth with 0.05% Tween, diluted, and plated on tryptic soy agar, bile esculin azide (PML microbiologicals, Wilsonville, OR), and McConkey plates to monitor decolonization of the intestinal flora.

After 10 days, mice were switched to drinking water supplemented with 125 mg/L cefoxitin and 100 mg/L metronidazole, as well as 5 × 10^7 ^cfu/ml bacteria from a fresh overnight culture. One group of eight mice received the wild-type strain T9; the other group of eight mice received the biofilm-negative transposon mutant 10D5. Both strains showed equal viability in water with the antibiotics mentioned above, which was tested by plating dilutions at days 0, 1, 2, and 3. The cages, water, and food were again changed every other day, and fecal pellets were collected, homogenized, diluted, and plated every or every other day. The colonization was documented as the number of bacteria per gram of stool per mouse.

## Authors' contributions

RC was involved in conception and design, acquisition of data, and analysis and intrepretation; SK was involved in acquisition of data, and analysis and intrepretation; FF was involved in acquisition of data, and analysis and intrepretation; LB was involved in acquisition of data, and analysis and intrepretation; JH was involved in conception and design, acquisition of data, and analysis and intrepretation;

## Figures and Tables

**Table 1:** Strains of *Enterococcus faecalis *used in the present study

**Strain**	**Manipulation**	**Phenotype**	**Reference**
EFS T9	wild-type	strong BF in glucose or maltose	[23]
EFS 10D5	*Tn917 *insertion into *bop*B	no BF in glucose-containing medium	[8]
EFS TDM	deletion of *bop*A, *bop*B, and *bop*C	strong BF in glucose-containing medium	[8]
